# INTERGENERATIONAL HAIKU-MAKING ACTIVITY: CREATING MEANINGFUL CONNECTIONS ACROSS GENERATIONS

**DOI:** 10.1093/geroni/igad104.1945

**Published:** 2023-12-21

**Authors:** Yoshiko Matsumoto, Harumi Maeda, Emily Wan, Deven Bansal, Paolo Tayag

**Affiliations:** Stanford University, Stanford, California, United States; Stanford University, Stanford, California, United States; Waseda University, Tokyo, Tokyo, Japan; Stanford University, Stanford, California, United States; Stanford University, Stanford, California, United States

## Abstract

Intergenerational activities rooted in creative arts can improve the social connectedness and quality of life of older people living with cognitive impairment (e.g., Dorris et al., 2022; Ihara et al., 2022). Our study further explores the potential of humanities-based approaches to improve well-being and create meaningful connections across generations. Addressing the increasing concerns of loneliness and social isolation among older people living with cognitive impairment, we carried out a haiku-making activity in which 2-3 older adults with mild cognitive impairment (MCI) interact with 2-3 university students in the creation of haiku poems based on seasonal photos. This Japanese poetic form can be composed using short phrases that describe emotional responses to natural scenes, free from the logical and grammatical constraints of prose. This structure enables a failure-free environment that engages participants in self-expression and imagination. Additionally, haiku’s simple but often unfamiliar format allows all participants to interact and contribute on an even playing field (Author et al., in press). Our analysis of the (1) recorded haiku-making activity sessions, (2) recorded post-session interviews, and (3) completed haiku poems reveals positive impacts of the activity on both the older participants with MCI and the younger participants. Our findings illustrate how haiku-making and haiku-based conversations promote emotional well-being and intergenerational understanding among participants. This study presents a model for how incorporating perspectives from the arts and humanities into gerontology/geriatrics programs can improve quality of life and enhance meaningful social/intergenerational connections across life stages.

